# Recent Advances in Replication and Infection of Human Parvovirus B19

**DOI:** 10.3389/fcimb.2018.00166

**Published:** 2018-06-05

**Authors:** Safder S. Ganaie, Jianming Qiu

**Affiliations:** Department of Microbiology, Molecular Genetics and Immunology, University of Kansas Medical Center, Kansas City, KS, United States

**Keywords:** parvovirus B19, human, erythroid precursor cells, infection, DNA replication, RNA processing

## Abstract

Parvovirus B19 (B19V) is pathogenic to humans and causes bone marrow failure diseases and various other inflammatory disorders. B19V infection exhibits high tropism for human erythroid progenitor cells (EPCs) in the bone marrow and fetal liver. The exclusive restriction of B19V replication to erythroid lineage cells is partly due to the expression of receptor and co-receptor(s) on the cell surface of human EPCs and partly depends on the intracellular factors essential for virus replication. We first summarize the latest developments in the viral entry process and the host cellular factors or pathways critical for B19V replication. We discuss the role of hypoxia, erythropoietin signaling and STAT5 activation in the virus replication. The B19V infection-induced DNA damage response (DDR) and cell cycle arrest at late S-phase are two key events that promote B19V replication. Lately, the virus infection causes G2 arrest, followed by the extensive cell death of EPCs that leads to anemia. We provide the current understanding of how B19V exploits the cellular resources and manipulate pathways for efficient virus replication. B19V encodes a single precursor mRNA (pre-mRNA), which undergoes alternate splicing and alternative polyadenylation to generate at least 12 different species of mRNA transcripts. The post-transcriptional processing of B19V pre-mRNA is tightly regulated through *cis-acting* elements and *trans-acting* factors flanking the splice donor or acceptor sites. Overall, in this review, we focus on the recent advances in the molecular virology and pathogenesis of B19V infection.

## Introduction

**Figure 1:**
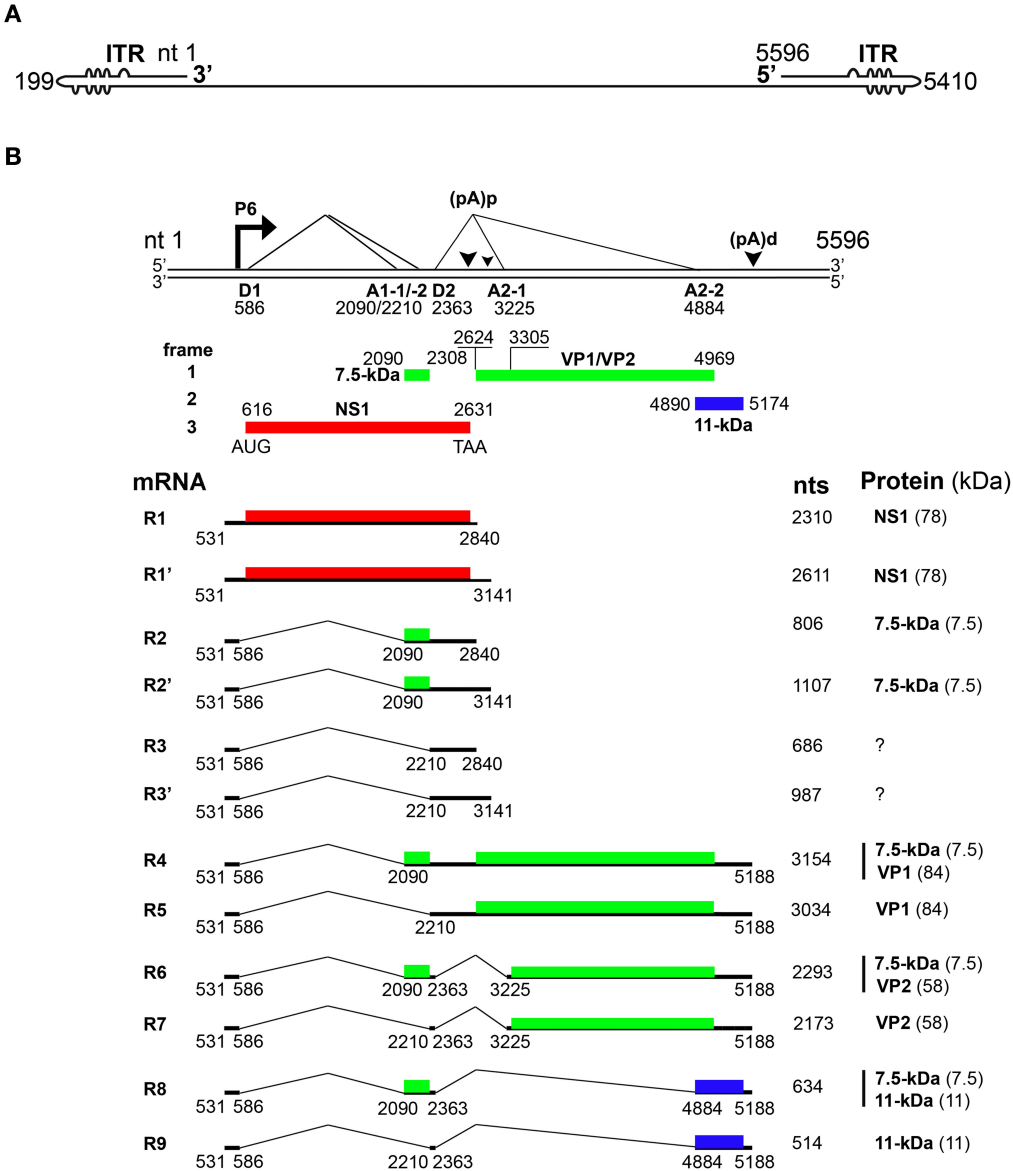
Transcription map of Parvovirus B19. **(A)** Linear ssDNA genome of B19V. The genome is flanked by two inverted terminal repeats (ITRs), containing unpaired and mismatched bases, shown as bulges and bubbles, respectively. **(B)** Double stranded replicative form of B19V genome. The viral promoter denoted as P6 transcribes a single precursor mRNA (pre-mRNA). Pre-mRNA has two donor sites (D1 and D2) and four acceptor sites (A1-1, A1-2, A2-1, and A2-2). Using alternative splicing and polyadenylation, pre-mRNA is processed into at least 12 different mRNAs (only R1–R9 shown here). Mature mRNAs polyadenylate at (pA)p or (pA)d sites. At least five different proteins are known to be encoded by different species of mRNA transcripts. Different colors indicate the use of different open reading frames for the translation of proteins. Question marks indicate mRNAs encoding unknown proteins.

Parvovirus B19 (B19V) is a small, non-enveloped virus that has a diameter of approximately 23–26 nm and contains a linear single-stranded DNA genome of 5.6 kb, flanked by two identical terminal hairpin structures (Figure 1A) (Qiu et al., 2017). B19V belongs to *Erythroparvovirus* of the *Parvoviridae* family (Cotmore et al., 2014). The name B19 was coined after the sample number containing the virus; panel B and no.19, during the screening of hepatitis B virus (Cossart et al., 1975). B19V infection causes several diseases in humans, including like fifth disease in children (Brass et al., 1982), transient aplastic crisis (Chorba et al., 1986), non-immune hydrops fetalis in pregnant women (Parilla et al., 1997), persistent anemia in immunocompromised patients, arthropathy (Hosszu and Sallai, 1997), cardiomyopathy (Simpson et al., 2016) and inflammation of various other tissues (Adamson-Small et al., 2014; Qiu et al., 2017).

In this review, we focus on recent advances in B19V tropism, viral DNA replication and viral transcription. Importantly, we will focus on four key factors involved in B19V replication that are: (a) erythropoietin (EPO) signaling; (b) hypoxia; (c) DNA damage response (DDR); and (d) late S-phase arrest. We also summarize the new advancements in B19V pre-mRNA processing and its regulation. We also discuss host factors STAT5 (Signal Transducer and Activator of Transcription-5) and RBM38 (RNA-Binding Motif protein-38), which regulate virus replication and mRNA processing, respectively. Lastly, we discuss the underlying mechanism of NS1 induced cell cycle arrest, B19V pathogenesis, and the future directions in the development of therapeutics for B19V infection.

## Viral entry and determinants of viral tropism

Productive infection of B19V is restricted to human erythroid progenitor cells, particularly, during the stages of burst forming unit-erythroid (BFU-E) to colony forming unit-erythroid (CFU-E) (Young et al., 1984a; Srivastava and Lu, 1988). B19V infects *ex vivo* expanded EPCs from human bone marrow (Young et al., 1984a; Ozawa et al., 1986; Srivastava and Lu, 1988), peripheral and umbilical blood (Serke et al., 1991; Schwarz et al., 1992; Sosa et al., 1992; Srivastava et al., 1992) and the fetal liver (Yaegashi et al., 1989; Morey and Fleming, 1992). In addition to expanded primary EPCs, various other cell lines have been used for B19V infection, including MB-02, UT7/Epo and UT7/Epo-S1 (megakaryoblastoid cell lines) (Shimomura et l., 1992; Munshi et al., 1993; Morita et al., 2001) and JK-1, KU812-Ep6 (erythroid leukemia cell lines) (Takahashi et al., 1993; Miyagawa et al., 1999). For efficient B19V replication, erythropoietin and hypoxia play a critical role under *in vitro* conditions (Chen et al., 2010a, 2011). B19V also infects endothelial cells of various tissues, but the infection is largely non-productive (Adamson-Small et al., 2014). In addition, U937 cells, circulating angiogenic cells (CACs) and CD34^+^ endothelial progenitor cells from bone marrow have also been reported to be susceptible to B19V infection (Munakata et al., 2006; Schmidt-Lucke et al., 2010, 2015). All these cells either express viral receptor/co-receptors or use an antibody dependent route via c1q receptor for viral entry (von Kietzell et al., 2014).

**Figure 2:**
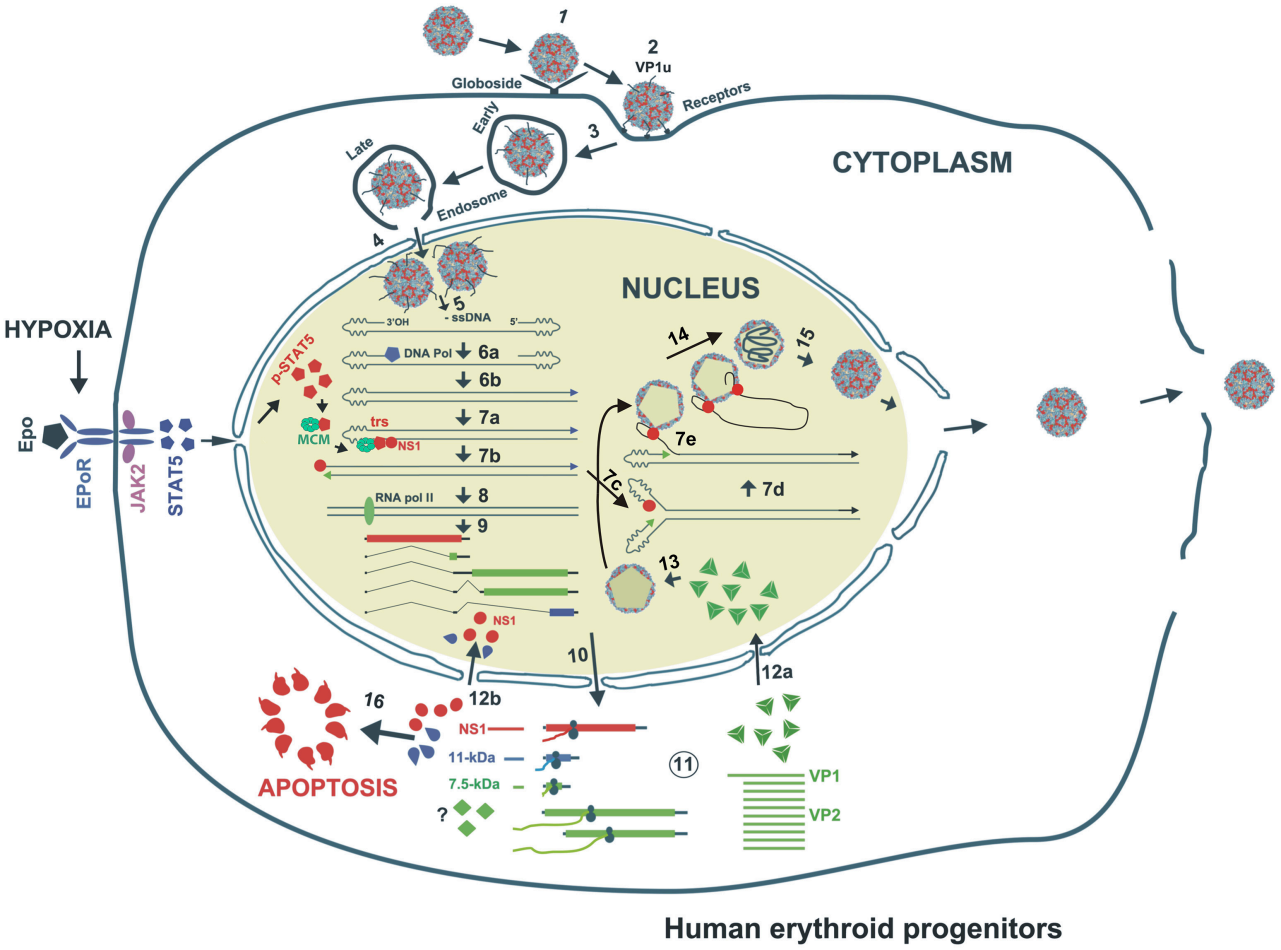
Proposed model of B19V life cycle. B19V infects human erythroid progenitor cells. The virus first interacts with globoside (Step 1) and undergoes a conformational change that exposes VP1u which subsequently binds an unknown co-receptor (Step 1). Thereupon, the virus is endocytosed and somehow escapes the lysosomal route and enters the nucleus (Step 3). Inside the nucleus, the virion uncoats and releases the ssDNA genome (Step 4). Using the 3′OH of the left ITR, the second strand is synthesized to form a functional origin of replication (Step 5). Next, EPO and hypoxia activates and increases pSTAT5, which interacts with MCM and then binds Ori region. NS1 binding to NS1BE is critical for nicking ssDNA at trs and for helicase activity (step 6). The nicking creates a new 3′ OH end to continue DNA replication that results into duplex replicative intermediate (Step 7). The dsDNA form also transcribes a single pre-mRNA that is processed into various mRNAs which are exported to cytoplasm for translation (Step 8). VP1/2 assemble into trimers to form capsids, which are transported back to the nucleus (Step 9). Through strand displacement, ssDNA is packaged into capsids, which probably requires NS1 (step 10). NS1 and 11-kDa in the cytoplasm induce apoptosis (Step 11). After multiplication, the virions are released though cell lysis.

The primary receptor for B19V is globoside or P antigen (Figure 2) (Brown et al., 1993). However, all P antigen expressing cells are not permissive to B19V (Weigel-Kelley et al., 2001). Various other co-receptors like Ku80 (Munakata et al., 2005), integrin α5β1 (Weigel-Kelley et al., 2003) and antibody-mediated B19V entry routes (von Kietzell et al., 2014) are presumed to be involved in B19V entry. Importantly, the B19V capsid binds its primary receptor, P antigen and undergoes a conformational change, exposing VP1u, a unique (273 aa) N-terminus of the VP1 capsid protein (Figure 2) (Ros et al., 2006; Bonsch et al., 2008, 2010). Since Ku80 and α5β1 integrin haven't been shown to interact with VP1u, it has been hypothesized that VP1u interacts with some unknown co-receptor for subsequent internalization. Further, the N-terminal 100 amino acids of VP1u are required for internalization, which implies that the PLA2 (phospholipase A2) activity of VP1u is not essential for viral entry (Leisi et al., 2013). Interestingly, the VP1u region (without capsid) is efficiently internalized by B19V-permissive cells (Leisi et al., 2016a,b), which suggests that primary interaction of B19V with P antigen is required only for externalization of VP1u. Mature RBCs also express P antigen and hence show primary attachment to B19V, but the virus is not internalized (Bonsch et al., 2008). It is presumed that this primary interaction may be responsible for systemic dissemination of the virus. Similar to other parvoviruses, B19V uses the endocytic pathway but escapes lysosomal degradation to enter the nucleus for subsequent replication, transcription and packaging (Figure 2) (Harbison et al., 2008; Quattrocchi et al., 2012). In conclusion, the B19V first interaction with the globoside leads to the externalization of VP1u, which then interacts with some unknown co-receptor for internalization of the virus.

## Gene expression, splicing, and its regulation

Upon entry of the virus into the nucleus, the B19V ssDNA genome is converted to double-stranded replicative form (dsRF), which acts a template for both DNA replication and transcription (Figure 2) (Cotmore and Tattersall, 2014). The dsRF viral DNA has a unique but single promoter at map unit 6 (P6) that expresses a single precursor mRNA (pre-mRNA) (Figure 1B) (Blundell et al., 1987; Doerig et al., 1987). The P6 promoter has an upstream enhancer region and NS1 binding elements that bind transcription factors (e.g., CREBP, GATA, Oct1 etc.) and NS1 protein, respectively, for promoter transactivation (Blundell and Astell, 1989; Liu et al., 1991; Momoeda et al., 1994a; Raab et al., 2001). B19V pre-mRNA undergoes alternative splicing and polyadenylation to express at least 12 mature mRNA transcripts that encode two structural (VP1 and VP2) and three non-structural (NS1, 7.5 and 11-kDa) proteins (Ozawa et al., 1987; Beard et al., 1989; Yoto et al., 2006). The pre-mRNA contains two splice donor sites (D1 and D2) and four acceptor sites (A1-1, A1-2, A2-1, and A2-2) (Figure 1B) (Qiu et al., 2017). In addition, it harbors two proximal [(pA)p]1/2 and a distal [p(pA)d] polyadenylation sites (Figure 1B) (Qiu et al., 2017). Unspliced mRNA transcripts (R1 and R1′) that polyadenylate at (pA)p encode the NS1 protein while those spliced at A1-1 (R2 and R2′) and use (pA)p sites encode the 7.5-kDa protein (Figure 1B). The mRNA transcripts polyadenylated at (pA)d and where 1st intron (R4 and R5), 1st and 2nd (R6 and R7), and 1st, 2nd, and 3rd introns (R8 and R9) are spliced out encode the VP1, VP2, and 11-kDa proteins, respectively (Figure 1B) (Luo and Astell, 1993; St Amand and Astell, 1993; Yoto et al., 2006). Whether other mRNAs (R3 and R3′) encode a protein or play any role during virus infection is unknown.

While the B19V mRNAs are splice products from a single pre-mRNA, the expression levels of the different encoded proteins varies considerably (Guan et al., 2011b). Therefore, the virus must employ different strategies to regulate the level of gene expression. In the absence of viral DNA replication, most of the mRNA transcripts polyadenylate at (pA)p leading to the expression of NS1 and 7.5-kDa proteins in both B19V-permisssive or non-permissive cells (Figure 1B) (Liu et al., 1992). Viral DNA replication facilitates the read-through of (pA)p and overcomes the blockade to express mRNAs that polyadenylate at (pA)d encoding for the VP1, VP2 and 11-kDa proteins (Guan et al., 2008). Thus, the early and late phases of virus infection are dominated by NS1-encoding and VP/11-kDa-encoding mRNAs, respectively (Bua et al., 2016). An alternative model for virus infection was also proposed suggesting that B19V genome be considered as single, two state replicative and transcription unit, where the increase in viral RNA correlates with viral DNA levels (Bonvicini et al., 2006). Next, the central exon or exon 2 (spanning A1-1/2 to D2) harbors serine arginine (SR) protein binding GAA motifs, and the GAA motif between A1-1 and A1-2, constitutes exon splicing enhancer 1 (ESE1), which defines exon-2 and facilitates splicing at A1-1. The 5′ end of exon 2 promotes splicing at A1-2 and serves as exon splicing enhancer 2 (ESE2) (Guan et al., 2011a). Splicing at second donor site (D2) is critical for the expression of capsid proteins and 11-kDa encoding mRNAs, and also competes with polyadenylation at (pA)p (Guan et al., 2011b). Binding of U1 snRNA to D2 splice donor site inhibits polyadenylation at (pA)p (Guan et al., 2011a). D2 is a weak splice donor site and requires two *cis-acting* elements: exon splicing enhancer 3 (ESE3) and intron splicing enhancer 2 (ISE2) for its efficient splicing (Guan et al., 2011a). Hence, the interplay of *cis-acting* elements and *trans-acting* factors determine the splicing efficiency of different splice sites to regulate the expression of different mRNA species. While looking for the *trans-acting* factors that bind ISE2, we demonstrate that RNA binding protein-RBM38, expressed in the middle stages of erythropoiesis, promotes the expression of 11-kDa protein (Ganaie et al., 2018). Specifically, RBM38 binds ISE2 and promotes splicing of third intron (D2 to A2-2), that results in the production of 11-kDa encoding mRNAs. Therefore, RBM38 is one of the essential *trans-acting* factors that regulates the expression of 11-kDa protein (Ganaie et al., 2018). In conclusion, B19V efficiently uses the alternative splicing and alternative polyadenylation processes to ensure the expression of the viral proteins at a given ratio. However, such tight regulation is dependent on the *cis-acting* elements and the *trans-acting* factors flanking the splice donor/acceptor sites.

## Viral proteins and their functions

B19V expresses three non-structural (NS1, 11- and 7.5-kDa) and two structural proteins (VP1 and VP2).

### Non-structural proteins

#### Non-structural protein 1 (NS1)

**Figure 3:**
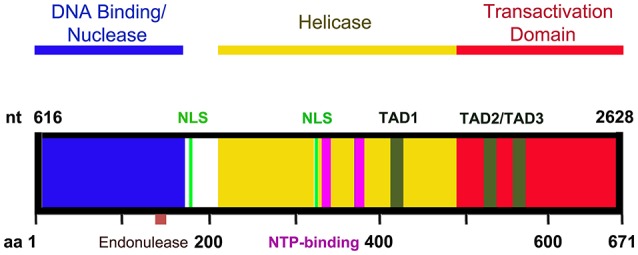
A diagram of NS1 functional domains. The N-terminus (amino acid 2-176) of NS1 possesses DNA binding and endonuclease activity. The endonuclease motif resides between amino acids 137 and 145. The central region of NS1 exhibits putative helicase activity. Transactivation activity is restricted to the C-terminus of NS1. NS1 carries two nuclear localizing signals, between amino acids 177–179 and 316–321 (NLS, in green). Three putative transactivation domains have been identified in the C-terminus of the NS1 protein: TAD1 (aa 416–424), TAD2 (aa 523–531), and TAD3 (aa 566–574). The central region also contains two NTP binding motifs between amino acids 323–378 and 367–378 (NTP binding motifs, in purple).

**Figure 4:**
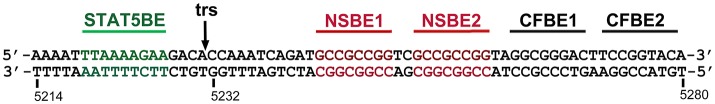
A diagram of the B19V minimal origin of viral DNA replication (Ori). B19V has a 67-bp long minimum origin of DNA replication (Ori) at each end of the genome. Ori harbors two NS1 binding elements (NSBE1&2, in red), one STAT5 binding element (STAT5BE, in green), a terminal resolution site (trs, black), and two potential cellular factor binding elements (CFBE1&2). Question marks denote two unidentified host factors binding Ori.

**Figure 5:**
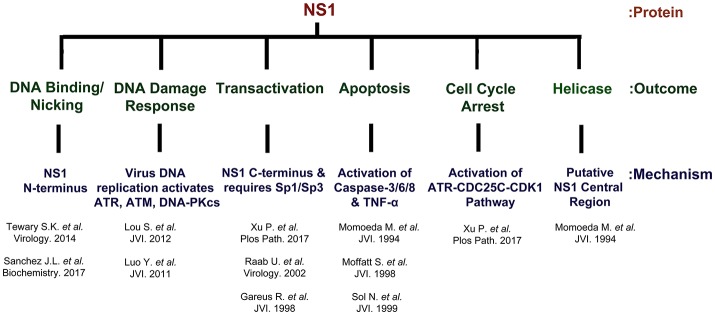
NS1 is a multi-functional protein. The B19V NS1 multimer binds the dsDNA form of the genome at NSBE1-2 via N-terminus region (5–7), but nicks ssDNA at trs and covalently attaches to the 5′ end. NS1 induces a DNA damage response that is essential for virus replication. The virus replication process leads to the activation of ATR, ATM and DNA-PKcs. However, the activation of ATR and DNA-PKcs, but not ATM, is essential for virus replication. NS1 transactivates its P6 promoter with the assistance of Sp1/Sp3. NS1 is a global transactivator and regulates ~1,700 genes. NS1 induces apoptosis through the activation of caspases 2/6/8 and TNF-α. The central region of NS1 protein exhibits putative helicase activity.

NS1 is 671 amino acid long protein that has a MW of ~78 kDa. NS1 contains two nuclear localization signals; KKPR (177–179) and KKCGKK (316–321) (Figure 3) and is found predominantly in the nucleus of infected cells (Cotmore et al., 1986; Ozawa and Young, 1987; Ozawa et al., 1987; Wan et al., 2010). NS1 contains a DNA binding and endonuclease domain at the N-terminus (Tewary et al., 2014), an ATPase and NTP-binding domains in the central region (Momoeda et al., 1994b) and a transactivation domain at its C-terminus (Lou et al., 2012) (Figure 3). NS1 is critical for virus DNA replication (Zhi et al., 2006), and binds NSBEs (NS1 binding elements) of the minimum origin of replication of B19V dsRF DNA (Figure 4) (Tewary et al., 2014). Upon NS1 binding to NSBEs, it presumably opens up the dsRF DNA and nicks the ssDNA substrate at trs (terminal resolution site) (Figure 2) (Sanchez et al., 2016). With the assistance of Sp1/Sp3, NS1 binds P6 promoter for its transactivation (Gareus et al., 1998; Raab et al., 2002). NS1 induces apoptosis via NTP-binding motif (328-335 amino acid) (Momoeda et al., 1994b; Moffatt et al., 1998; Poole et al., 2006), a DNA damage response (Luo et al., 2011; Lou et al., 2012) and cell cycle arrest (Morita et al., 2003; Wan et al., 2010; Luo et al., 2013; Xu et al., 2017). The putative transactivation domain 2 (TAD2) of NS1 is critical for cell cycle arrest and the transactivation of host genes. NS1 is thought to be a global transactivator as the expression of NS1 protein in UT7/Epo-S1 affected around 1,770 genes, by upregulating 1,064 genes and downregulating 706 genes (Xu et al., 2017). In short, NS1 is a multifunctional protein and plays various roles during B19V infection (Figure 5).

#### 11-kDa protein

The 11-kDa protein is expressed at high levels during B19V infection and localizes more in the cytoplasm than in the nucleus of infected cells. The abundance of the 11-kDa protein in infected cells is at least 100 times greater than NS1 protein (St Amand and Astell, 1993; Chen et al., 2010b). The 11-kDa protein is potent inducer of apoptosis during B19V infection and involves caspase-10 (Figure 2) (Chen et al., 2010b). We demonstrate that the 11-kDa protein enhances viral DNA replication (~10-fold), and thus determines virion production (Ganaie et al., 2018). Finally, the 11-kDa protein has also been implicated in VP2 production and its distribution (Zhi et al., 2006).

#### 7.5-kDa protein

The function(s) of 7.5-kDa are still unknown.

### Structural proteins

#### Capsid proteins (VP1 and VP2)

VP1 is a minor capsid protein, 781 amino acids long (~84 kDa) (Ozawa and Young, 1987). VP1 shares the same C-terminus with VP2, with extra 273 amino acids, called as VP1-unique (VP1u) (Ozawa and Young, 1987; Ozawa et al., 1987). VP2 is a major capsid protein, 554 amino acid long (~58 kDa) (Ozawa and Young, 1987). VP1 expression is low (Ozawa et al., 1988) and with VP2 assembles into the viral capsid (VP1:VP2 = 1:20) (Figure 2) (Ozawa and Young, 1987). VP2 has a nuclear localization signal at its C-terminus, therefore both the VP1 and VP2 proteins are found in the nucleus of infected cells (Pillet et al., 2003). The capsid first interacts with P antigen (Figure 2) (Brown et al., 1993) and thereafter first 100 amino acids of VP1u help in internalization of the virus particles (Leisi et al., 2013). VP1u region from 128 to 160 amino acids exhibits phospholipase A2 activity (Dorsch et al., 2001, 2002; Zádori et al., 2001), which is possibly used to evade lysosomal fusion and ensure nuclear entry of the virions. In conclusion, the viral structural proteins (VP1/VP2) form the viral capsids for viral DNA encapsidation, whereas, non-structural proteins ensure the efficient virus replication, packaging and release of infectious viral particles. The non-structural proteins need to be further investigated for their functional characterization.

## B19V replication and its regulation

B19V replication takes place in the nucleus of the infected cells. The single-stranded genome of the virus is first converted to dsRF DNA. B19V genome (RF) contains a 67-bp (nt 5214–5280) long minimum origin of replication (Ori) at each ends (Guan et al., 2009). Although the origins function independently, but both origins are required for the efficient DNA replication of the virus (Ganaie et al., 2017). B19V genome can replicate even with half inverted terminal repeats (ITR), however, the full ITRs significantly enhance viral DNA replication (Ganaie et al., 2017). Ori contains NS1 binding elements (NSBEs) (Tewary et al., 2014), terminal resolution site (trs) (Sanchez et al., 2016), STAT5 binding site, and potential host factor binding sites (Figure 4) (Ganaie et al., 2017). NS1 binds NSBEs and nicks DNA at trs (Sanchez et al., 2016), whereas STAT5 binds Ori and recruits the MCM (Minichromosome Maintenance) complex (Ganaie et al., 2017). After NS1 nicking, DNA replication continues, and presumably follows rolling hairpin model of replication (Figure 2), as suggested for other parvoviruses (Tattersall et al., 2005). Upon microarray analysis of the dynamic transcriptome of B19V infected EPCs, we recently found that DNA metabolism, DNA replication, DNA repair, DNA damage response, cell cycle and cell cycle arrest pathways are significantly altered upon virus infection (Zou et al., 2017). Broadly, B19V replication is regulated by the following factors:

### EPO-signaling

In response to low oxygen tension, human kidney interstitial fibroblasts secrete EPO, a glycoprotein cytokine that promotes the differentiation and development of erythroid progenitors that results in the production of mature RBCs (Testa, 2004). During erythropoiesis, pluripotent hematopoietic stem cells (HSCs, CD34^+^) are differentiated into enucleated erythrocytes, encompassing the following stages – BFU-Es, CFU-Es, normoblasts, erythroblasts, reticulocytes and finally the mature erythrocytes (Ogawa, 1993; Testa, 2004). The earlier stages of differentiation are EPO-independent (Ogawa, 1993), and rely on factors like, stem cell factor (SCF) (Dai et al., 1991; Papayannopoulou et al., 1993), IL-6 (Sui et al., 1996), and IL-3 (Papayannopoulou et al., 1993). However, the late stage differentiation process requires EPO (Koury and Bondurant, 1992; Ogawa, 1993). The exclusive tropism of B19V for erythroid progenitor cells partly depends on the expression of receptor and co-receptors on the cell surface and partly on the essential host cellular factors for efficient virus replication. Erythroid lineage cells depend on EPO for survival (Grebien et al., 2008), but B19V also needs EPO for its replication (Chen et al., 2010a). BFU-Es and CFU-Es, the late stage erythroids, are highly susceptible to B19V infection (Takahashi et al., 1990). B19V-semi permissive cell lines (e.g., UT7/Epo-S1) depend on EPO for cell proliferation and survival. Interestingly, B19V-permissivity of these cell lines strictly depends on EPO (Figure 2) (Takahashi et al., 1990). CD36^+^ EPCs differentiated from CD34^+^ in the absence of EPO are not permissive to B19V infection and B19V genome replicates only in the presence of EPO and requires phosphorylated Janus kinase 2 (JAK2) (Chen et al., 2010a). EPO binds EPO-receptor and activates ERK, Phosphoinositide 3-kinase (PI3K) and JAK2-STAT5 pathways (Figure 2) (Lodish et al., 2009). JAK2-STAT5 pathway is essential for B19V replication while the ERK pathway negatively regulates B19V replication and PI3K is dispensable for virus replication (Chen et al., 2011). Although EPO signaling activates the ERK pathway, B19V inhibits the ERK pathway presumably through its 11-kDa protein (Fan et al., 2001; Chen et al., 2011). Therefore, B19V fine tunes the EPO-signaling that favors efficient virus replication. In order to understand the underlying mechanism of STAT5 dependent B19V replication, we demonstrate that STAT5 phosphorylation is critical for B19V DNA replication (Ganaie et al., 2017). The viral Ori harbors a STAT5 binding site (Figure 4) and phosphorylated STAT5 binds viral origins both *in-vivo* as well as *in-vitro* (Ganaie et al., 2017). The mutation of STAT5 binding site within viral origins completely abolishes viral DNA replication (Ganaie et al., 2017). Furthermore, STAT5 interacts with the MCM complex and the disruption of STAT5-Ori complex leads to decrease in virus DNA replication and the abundance of MCM complex decreases significantly at the viral Ori (Ganaie et al., 2017). Therefore, in our proposed model, B19V utilizes the STAT5 interaction with viral Ori to recruit the MCM complex for initiation of viral DNA replication (Ganaie et al., 2017).

### Hypoxia

During the propagation of B19V in *ex-vivo* expanded CD36^+^ EPCs, progeny virion production is not as efficient as under natural conditions in human bone marrow on B19V infection of EPCs. The viremia in B19V infected patients goes up to 10^13^ genomic copies per ml of plasma (Wong and Brown, 2006; Takano and Yamada, 2007), which indicates the requirement of other factors in determining the production of virions. Oxygen tension is low in bone marrow (Rogers et al., 2008) and lower oxygen pressure favors erythroid cell development in culture (Koller et al., 1992). Interestingly, B19V infected human EPCs at hypoxia (1% O_2_) enhances viral gene expression, viral replication, and virus production. Hypoxia augmentation of B19V replication is independent of the PHD/HIFα pathway. Hypoxia also enables the B19V-infected pluripotent erythroid cells (KU812F) to yield high progeny virions (Caillet-Fauquet et al., 2004). Increase in the productive B19V infection under hypoxic conditions wasn't due to increase in the B19V entry or intracellular trafficking of the virus. Also, HIF-1 was shown not to play any role in hypoxia-induced enhancement in B19V infectivity (Chen et al., 2011). However, hypoxia regulates the EPO/EPO-receptor signaling pathway by upregulating STAT5 and downregulating MEK activation, thereby enhancing B19V DNA replication in both B19V-infected EPCs and M20 (infectious clone)-transfected UT7/Epo-S1cells (Chen et al., 2011). We strongly believe that upregulation of STAT5 and downregulation of MEK/ERK signaling during hypoxia promotes virus replication. The B19V infectious clone grows rapidly (~80 times) in UT7/Epo-S1 cells under hypoxia (Chen et al., 2011). The culture of EPCs or UT7/Epo-S1 cells under hypoxia for B19V propagation is currently the best culture system in use. The efficient replication of M20 infectious clone and the subsequent production of infectious virions holds promise to study B19V replication and the underlying mechanism(s) of replication. Also, mutagenesis of B19V molecular clone M20 could help to understand the role of individual viral proteins and the specific protein domains in virus replication.

### Late S phase cell cycle

B19V DNA replication is dependent on host cellular DNA replication, since the virus doesn't encode a viral DNA polymerase (Luo and Qiu, 2015). B19V induces cell cycle arrest in both virus-infected CD36^+^ EPCs and UT7/Epo-S1 or M20 transfected UT7/Epo-S1 cells at G2/M (Morita et al., 2001; Luo et al., 2013). Virus infected UT7/Epo-S1-arrested cells display 4N DNA content and (5′-bromo-2-deoxy-uridine) BrdU-pulse labeling for *de novo* DNA synthesis along with DAPI staining indicates that the cells are precisely in late-S phase (Luo et al., 2013). Upon B19V infection of UT7/Epo-S1, cyclin A, cyclin B1 and phosphorylated cell division cycle 2 (CDC2) were shown to accumulate and CDC2-cyclin B1 complex displayed enhanced kinase activity (Morita et al., 2001). The sequestration of cyclin B1 to the cytoplasm in B19V-infected cells indicates that B19V somehow prevents its import to nucleus, thus results into cell cycle arrest at G2 phase (Morita et al., 2001). NS1 alone has been shown to induce a true G2/M cell cycle arrest, doesn't show BrdU incorporation but displays 4N DNA content (Luo et al., 2013), and de-regulation of E2F family transcription factors have been implicated for such arrest (Wan et al., 2010).

While B19V induces DDR that promotes virus replication, virus-induced DDR is not involved in cell cycle arrest at G2/M (Lou et al., 2012). Like other autonomous parvoviruses, B19V infection also induces arrest at S phase (Luo et al., 2011, 2013). Therefore, B19V induced late S phase arrest is possibly an outcome of replication-induced S phase arrest and NS1-induced G2/M arrest. Various S phase replication factors like polymerase delta (pol δ), proliferating cell nuclear antigen (PCNA), replication factor C-subunit 1 (RFC-1), the MCM complex except the DNA repair DNA polymerases are actively recruited to viral replication centers with pol δ and pol α essential for viral DNA replication (Luo et al., 2013; Zou et al., 2017). It is likely that like other parvoviruses (Parris and Bates, 1976; Oleksiewicz and Alexandersen, 1997; Deleu et al., 1999), B19V exploits the cell cycle arrest at late S phase and uses S phase factors to for viral DNA replication. In addition, replication protein A-32 (RPA32) also colocalizes with viral replicating machinery. Although phosphorylated RPA32 forms also show colocalization, phosphorylation itself seems dispensable for virus replication (Zou et al., 2017), supporting the observation that B19V uses S phase for genome amplification.

### DNA damage response (DDR)

DNA damage response (DDR) is a cellular defense mechanism to preserve genomic stability and integrity in response to double strand breaks (DSBs), single strand DNA breaks, or installed replication (Ciccia and Elledge, 2010). There are three major kinases (mediators) responsible for signaling downstream DDR effects, including Ataxia telangiectasia mutated (ATM), Ataxia telangiectasia and Rad3 related (ATR), and DNA dependent protein kinase, catalytic subunit (DNA-PKcs). Apart from recognizing the damaged cellular DNA, DDR is also activated by various DNA viruses either to combat the infection by invoking innate immune response or to facilitate viral DNA replication (Luo and Qiu, 2015; Trigg and Ferguson, 2015). B19V infection induces a DDR by activating all the three PI3K kinases (ATR, ATM, and DNA-PKcs) (Luo et al., 2011). Phosphorylated ATM, ATR, DNA-PKcs and their downstream effectors (CHK1, CHK2, and Ku70/80) localize within the virus replication centers. Activated ATR and DNA-PKcs, but not ATM were found essential for B19V replication (Luo et al., 2011; Lou et al., 2012). Further, it was found that mere expression of the viral genes didn't lead to the phosphorylation of RPA32 and γH2AX, a hallmark of DDR (Lou et al., 2012). Interestingly, NS1 itself phosphorylates ATR to induces cell cycle arrest, however, such activation of ATR doesn't lead to phosphorylation of RPA32 and γH2AX (Xu et al., 2017). It was found that replicating, infectious clone-M20, but not a replication deficient M20 mutant, led to the induction of DDR (Lou et al., 2012), which implies that the replication process *per se* is responsible for inducing DDR.

In conclusion, Epo signaling and the virus induced- DDR and late S-phase arrest are essential for the efficient virus replication. Hypoxia stimulates virus replication through upregulation of the STAT5A. These findings have revealed the new mechanistic insights into B19V replication and also helped in the identification of novel targets for inhibiting B19V at the replication level. However, It remains to be seen that how virus infection-induced DDR promotes the efficient B19V replication in the erythroid cells?

## Viral pathogenesis

### Productive infection of B19V induces cell cycle arrest and erythroid cell death

B19V infection induces cell cycle arrest at G2 phase (Morita et al., 2001). Upon further analyses of the cell cycle during the virus infection, it was found that the arrest at G2 phase has 4N DNA content but also incorporates BrdU, a thymidine analog, suggesting that the infected cells are in late S phase (Luo et al., 2013). During early infection, the cells are precisely at late S phase, however, at the late phase of infection most infected cells are found in G2 phase (Luo et al., 2013). Interestingly, the expression of NS1 induces a true G2 arrest where cells don't incorporate BrdU but only exhibit 4N DNA content (Luo et al., 2013). There are many players responsible for inducing cell cycle arrest during B19V infection. During the B19V induced cell cycle arrest of UT7/Epo-S1 cells, it was observed that nuclear import of CDC2/cyclin B1 is prevented (Morita et al., 2001). NS1 itself causes a true G2 arrest by importing the repressive E2F transcription factors (E2F4/E2F5) (Wan et al., 2010). A putative NS1 transactivation domain-2 (TAD2) was found responsible for NS1-induced G2 arrest (Lou et al., 2012). Recently, we explored the underlying mechanism of the NS1 induced cell cycle arrest in great detail (Xu et al., 2017). NS1-TAD2 domain transactivates several host genes that lead to the activation of ATR. Activated ATR phosphorylates cell division cycle 25C (CDC25C) at serine-216 through the activation of CHK1 (Xu et al., 2017). Phosphorylated CDC25C at S216 reduces its phosphatase activity and renders it complexed with 14-3-3 protein in the cytoplasm (Peng et al., 1998). As a result, inactive CDC25C is unable to dephosphorylate cyclin B1/CDK1 (pT14/Y15) complex to activate it. An active cyclin B1/CDK1 complex is essential for G2 to M transition (Dunphy et al., 1988). In NS1-expressing UT7/Epo-S1 cells, nuclear entry of cyclin B1/CDK1 complex is not hampered, rather the complex exhibits reduced kinase activity (Xu et al., 2017). Hence, B19V NS1 induces G2 arrest by activating the ATR-CDC25C-CDK1 pathway (Xu et al., 2017). NS1 activation of ATR doesn't lead to the activation of γH2AX and RPA32, a hallmark of DDR (Lou et al., 2012; Xu et al., 2017). Moreover, DNA replication induced DDR and thereafter, activation of ATR leads to the arrest of cells at late S phase (Luo et al., 2013). It appears that NS1 or DNA replication mediated activation of ATR transduce signaling through different downstream pathways and results in cells arrested at different phases of cell cycle. The last factor implicated in B19V infection induced cell cycle arrest is the viral genome itself. A nucleotide sequence 5′-GTTTTG T-3′ from the viral promoter region arrests BFU-E progenitor cells at S and G2/M phase (Guo et al., 2010). This promoter sequence is a CpG oligodoxynucleotide-2006 analog that is a ligand of toll-like receptor 9 (TLR9). It appears that viral genomic replication initially stalls infected cells at late S phase and later with the help of NS1 arrests at G2/M, which eventually leads to cell death. B19V infection specifically targets BFU-E and CFU-E progenitors (Mortimer et al., 1983), disrupts erythropoiesis and results in the transient aplastic crisis (Young et al., 1984b). The virus-induced cell death is apoptotic in nature and involves caspase-3/6/8 activation (Moffatt et al., 1998; Sol et al., 1999). B19V NS1 activates the extrinsic apoptotic pathway involving the TNF-α pathway in CD36^+^ EPCs or UT7/Epo cells (Sol et al., 1999). The B19V encoded 11-kDa protein is also implicated in causing cell death through apoptosis, which involves caspase-10 (Chen et al., 2010b). It was found that 11-kDa is a more potent inducer of apoptosis than NS1 (Chen et al., 2010b).

### Non-productive infection of B19V causes inflammatory diseases of various tissues

Virus infection is seen in non-erythroid cells as well. The virus uses an alternative entry route by complexing with antibody and entering through complement factor C1q and C1q receptor mediated endocytosis (von Kietzell et al., 2014). There is no clear evidence that B19V replicates or produces virions in any non-erythroid cell lineage, hence the infection is considered largely non-productive.

B19V predominantly infects endothelial cells of various tissues (e.g., aorta, umbilical vein, and pulmonary arteria etc.) (von Kietzell et al., 2014). Other cell lines infected include U937 cells (Munakata et al., 2006), circulatory angiogenic cells (CACs) and CD34^+^KDR^+^ endothelial progenitor cells (Schmidt-Lucke et al., 2010, 2015). B19V infection appears to be persistent and viral genes are silenced through methylation of CpG sites on the DNA (Bonvicini et al., 2012). Even after the infection is resolved, the viral DNA can be found in various tissues like spleen, liver, tonsils, testes and brain (Kerr, 2000; Adamson-Small et al., 2014).

Upon infection, the infected tissue evokes host-cellular response against the virus which culminates in a myriad of pathologies. B19V infection has been linked to several inflammatory diseases like cardiomyopathy (Simpson et al., 2016), rheumatoid arthritis (Simpson et al., 1984), hepatitis (Longo et al., 1998), vasculitis (Finkel et al., 1994), meningoencephalitis (Adamson-Small et al., 2014; Qiu et al., 2017). B19V infection or the expression of viral proteins can modulate the immune response. NS1 upregulates IFNAR1 and IL-2 (inflammatory response) and downregulates OAS1 and TYK2 (antiviral response) through the activation of STAT3/PIAS3 signaling pathway in human endothelial cells (HMEC-1) (Duechting et al., 2008). The increase in the expression of inflammatory molecules like NF-κB, IL-6 and COX2 correlated with expression of viral capsid proteins in the colon, thyroid and synoviocytes (Lu et al., 2006; Li et al., 2007; Wang et al., 2008; Polcz et al., 2013). Particularly, in synoviocytes, VP1u phospholipase A2 activity is implicated in the production of inflammatory response (Lu et al., 2006). NS1 induces apoptosis in hepatocytes through the activation of caspse-3 and caspase-9 (Poole et al., 2004, 2006).

In conclusion, the B19V productive infection causes cell death of the erythroid progenitors and the non-productive infection of non-erythroid tissues evokes inflammatory responses that leads to various pathologies. However, with the exception of the placental endothelium (Pasquinelli et al., 2009), no other non-erythroid cell type supports B19V multiplication.

## Conclusion and future directions

It has been reported that almost 40–60% of the world's population is infected with parvovirus B19 (Nunoue et al., 1985). However, such persistent infection is at sub-immunogenic levels, as viral load is kept under control by our immune system (Anderson et al., 1986; Kurtzman et al., 1989), particularly by neutralizing antibodies against B19V VP1u region (Anderson et al., 1995). During times when patients are under immunosuppression or during infection with other pathogens, the viral load increases, causing extensive cell death of erythroid progenitor cells and leads to various inflammatory diseases as described above (Heegaard and Brown, 2002; Qiu et al., 2017).

There is no specific treatment for B19V infection, except IVIG treatment (Watanabe and Kawashima, 2015) or blood transfusion (Soothill, 1990), so there is a need to develop antivirals for B19V infection. The new advancements in the field of B19V viral replication have identified various critical steps during the process of virus replication. One such important step is NS1 binding to the viral origin and subsequent nicking of B19V DNA at trs (Tewary et al., 2014). Recently, an *in-vitro* nicking assay for NS1 was developed (Sanchez et al., 2016), which could be utilized to screen for inhibitors of NS1 nicking. Furthermore, since the VP1u region is essential for viral entry and therefore, the peptide analogs of VP1u (1-100) (Leisi et al., 2013) or neutralizing monoclonal antibodies (Gigler et al., 1999) against VP1u can be employed to check B19V entry. We have demonstrated that STAT5 phosphorylation is essential for B19V DNA replication (Ganaie et al., 2017). Pimozide, a STAT5 inhibitor and an FDA approved drug abolishes virus replication in CD36^+^ EPCs (Ganaie et al., 2017). However the levels used in patients are too low to inhibit B19V. Therefore, derivatives of pimozide could be explored as potential drugs for B19V infection and for B19 related pathologies and included in prophylactic antivirals for transplant recipients. However, the development of new antivirals against B19V infection needs an animal model to validate any new treatment. Currently, simian parvovirus (SPV) infection of cynomolgus monkeys (O'Sullivan et al., 1994) is an alternative to screen anti-B19 antivirals in animal model system.

## Author contributions

All authors listed have made a substantial, direct and intellectual contribution to the work, and approved it for publication.

### Conflict of interest statement

The authors declare that the research was conducted in the absence of any commercial or financial relationships that could be construed as a potential conflict of interest.
